# Domestic violence and its associated factors among married women of a village development committee of rural Nepal

**DOI:** 10.1186/s13104-016-1986-6

**Published:** 2016-03-19

**Authors:** Diksha Sapkota, Sailesh Bhattarai, Dharanidhar Baral, Paras K. Pokharel

**Affiliations:** Kathmandu University School of Medical Sciences, Dhulikhel, Nepal; BPKIHS, Dharan, Nepal

**Keywords:** Controlling behavior, Domestic violence, Mental health, Occurrence

## Abstract

**Background:**

Violence against women is a global public health problem occurring in multitude of contexts and domestic violence is considered to be the most pervasive one. Poor enforcement of policies, limitation of researches and expertise in this field largely accounts for persistence of this problem and nature of domestic violence and its associated factors are poorly understood.

**Objectives:**

This research aimed to estimate the magnitude of different forms of domestic violence and identify its associated factors.

**Methods:**

Community based cross sectional study was conducted among 355 married women of reproductive age group of Kusheshwor, Sindhuli, Nepal. The questionnaire adapted from the World Health Organization Multi-Country Study was used for the face to face interviews. Occurrence of current domestic violence was used as outcome variable in logistic regression. Descriptive and multivariate analysis were performed in order to assess the magnitude of domestic violence and to identify its associated factors respectively.

**Results:**

Self-reported lifetime prevalence of physical violence was 29.6 % and past year prevalence was 15.2 %, while corresponding figures for sexual violence were 6.8 and 2.3 %, and for psychological violence were 31.0 and 18.3 %. Lifetime domestic violence was 38.6 % while in past 12 months, prevalence was 23.1 %. Furthermore, 12.4 % of women were experiencing all forms of violence concurrently. Women with controlling husband and having poor mental health were found to be at higher risk of domestic violence.

**Conclusions:**

Domestic violence is still rampant in our society with several forms of violence occurring together. In a country like Nepal, differentials power in relationship and poor mental health was found to be positively associated with violent episodes. This study highlights the infringement of women rights which can be the cause for serious public health consequences.

## Background

Violence against women (VAW) is a global issue with consequences in all dimensions of women’s health. Though violence occurs in different forms and settings, ‘domestic violence’ (DV) is considered as the most pervasive form [1]. It includes violence perpetrated by spouse, family members, and manifested through physical, sexual, psychological, economic abuse [2].

Globally, it has been a subject of research interest since 1970s but the detailed data indices is low in developing world [3]. Gradually, VAW is considered as a legitimate human right issue as well as a significant threat to women’s health and well-being [4]. World Health Organization (WHO) multi-country study reported that 15–71 % of women had experienced violence [5, 6]. According to Nepal Demographic Health Survey (NDHS) 2011, overall 1/3rd of women of reproductive age group had ever experienced violence and 17 % reported violence in past 12 months [7]. A study conducted in rural districts of Nepal revealed that almost half of the women (48 %) had experienced violence at some time in their lives, and 28 % had experienced violence in the past 12 months; where emotional violence (40.4 %) was most commonly reported type of violence followed by physical violence (26.8 %), sexual violence (15.3 %), and economic abuse/violence (8 %) [8]. Similarly, according to findings of a study from Nepal, overall 58 % of women had experienced some forms of sexual coercion from their husbands [9]. Approximately 28.6 % of women complained about several physical health problems followed by reproductive (28.2 %) and mental health problems (16.3 %) [10]. The context of gender based violence (GBV), driven by social, cultural, religious, and gender norms, is compounded by years of political conflict resulting the risk increment of violence [11]. Inclusion of module of DV for the first time in NDHS highlights it as a serious national issue with limited data related to it. DV exists in ‘culture of silence’ and is typically enmeshed in several factors operating at different levels of society [12–14]. On account of previous studies in Nepal several factors like economic dependency, marriage practices, alcoholic husband, illiteracy, female subservience, women’s lack of autonomy to name a few has been identified as aggravating factors of violence [8, 15, 16]. Wide ranging figures in different settings with various possible risk and protective factors highlight the need of continuous research approach on VAW in different cultures and circumstances [17]. It is sad to mention that the act against DV has not been implemented adequately in the real world as violence is still persisting in our society.

Several literatures have shaded light on the DV affecting the various domains of women’s life. Thus, the importance of knowledge of the prevalence and its associated factors of violence has been emphasized through these literatures in order to establish a holistic health environment. There are few literatures in our context that assess the mental health status of women and inspect its association with the occurrence of violent incidents. This study strive for fulfillment of the gaps in identifying need of restoring sound mental health of women in dealing DV effectively by providing evidence for the relationship between DV and its contributing factors including mental health status of women. Moreover, this study is believed to enlighten the existing condition of DV in a particular rural area of Nepal, which will supplement the existing literatures, assist the policy makers for planning and implementing appropriate interventions through identifying the associated factors.

## Methods

### Study design and study area

Jica has been implementing mediation programs against GBV through the Ministry of Federal Affairs and Local Development (MoFALD) based on community mobilisers at the Village Development Committee (VDC) level in two districts, Sindhuli and Mahottari since 2010 [18]. Kusheshwor VDC of Sindhuli district was randomly selected that accounts the practicability issues such as time, money and so on. Additionally, it comprises multi-ethnic groups including indigenous people popularly known as Hayu. Community based analytical cross sectional study was executed using the ecological model proposed by WHO for understanding the nature and associated factors of violence [3, 19]. This preliminary research study is limited to a particular region of Nepal, but similar representation of violence can be assumed to other rural areas of Nepal and this research isn’t meant to be representative rather supplementing to the literatures on DV.

### Sample size calculation

The calculation of sample size was manually done using formula.

$$n = \left( Z \right)^{2} {{pq} \mathord{\left/ {\vphantom {{pq} {L^{2} }}} \right. \kern-0pt} {L^{2} }}$$

In the above equation, Z value is 1.96 at 95 % confidence interval (CI), p is prevalence of DV which is taken as 31.5 % [6], q is complement of p and L is absolute precision whose standard value used for the calculation is 0.05. From the calculation, the sample size was found to be 331. In order to address non-response rate, sample size was increased by 10 % and it came to be 365. Further, the evaluated sample size was ratified using nMaster 2.0 software.

### Sampling procedure

Sampling unit was household and sampling frame was prepared with the name of the head of the household, obtained from the voter list of the Sindhuli VDC profile, 2068B.S (2011/12 A.D.). Systematic random sampling with an interval of three was employed to meet the required sample size. Thus, the eligible women meeting the inclusion criteria i.e. married for at least 2 years and staying with husband for last 6 months were interviewed from one in every third household. In absence of eligible respondent in the particular household, the following house was visited for the survey. Incase of more than one eligible respondent in a sampled household, random selection was made to further the interview process by lottery method.

### Data collection

Most of the questions were based on standard questionnaire adapted from WHO multi-country study on women’s health and DV with minor modifications. It consists of several open and close ended questions incorporated in self-constructed questionnaire to estimate the occurrence of violence and identify various associated factors [20, 21]. Self Reporting Questionnaire (SRQ)-20 developed by WHO collaborative study on mental health care was used to assess the mental health status of respondents [17, 21]. The questionnaire were translated into Nepali and then retranslated into English to ensure that the originality and meaning was retained. Pre-testing of the questionnaire with married women of reproductive age group in adjacent VDCs was completed and necessary amendments in the questionnaires were incorporated.

Data collection was accomplished within the limited period of 3 months (September, 2012 to December 2012). The one to one structured interview was conducted by the researcher herself and the location for the interview was selected on the basis of the respondent’s preference like at the fields, mills tap etc. in the absence of the third party. The house of the respondent was visited twice before moving on to the next house in case the respondent was out of reach at initial visit. However, altogether 370 women were approached for the interview, only 355 completed the interview. Hence, the survey ended up with only 355 women for the final data analysis having the response rate of 96 %.

The ethical clearance was obtained from Institutional Ethical Review Board (IERB) of BP Koirala Institute of Health Sciences (BPKIHS), Dharan. Informed (verbal) consent was taken from women for participation. The respondents were explicitly explained the motive and nature of survey as well as made aware of the termination of the interview on their own will at any point during the interview process without giving any reasons. Confidentiality and anonymity of any individual was not violated throughout the survey. This study was completed in 6 months starting from September 2012 to February 2013.

When victims of DV were encountered, after interview brochures were circulated in order to aware them about the DV (offence and punishment) Act 2009 AD and minimize such violence in near future. The victims were educated and referred to the community mediation center and appropriate service outlets for any assistance to tackle violence.

### Data management and analysis

#### Outcome variables

Different types of violence were assessed as:

##### Physical violence

1. Pushed her, shook her, or threw something at her.
2. Slapped her.
3. Twisted her arm or pulled her hair.
4. Punched her with his fist or with something that could hurt her.
5. Kicked her, dragged her, or beat her up.
6. Tried to choke her or burn her on purpose.
7. Threatened her or attacked her with a knife, gun, or any other weapon.
8. (If any one of the above acts is present, then it is considered that there is physical violence).

##### Sexual violence

1. Physically forced her to have sexual intercourse even when she didn’t want to.
2. Forced her to perform any sexual acts she didn’t want to.
3. Any degrading or humiliating sexual act.

(If any one of the above acts is present, then it is considered that there is sexual violence).

##### Emotional violence

1. Said or did something to humiliate her in front of others.
2. Threatened to hurt or harm her or someone close to her.
3. Insulted her or made her feel bad about herself.

(If any one of the above acts is present, then it is considered that there is emotional violence).

[If woman (as a victim) gives a positive response to any of the questions in a set, it was considered to indicate the occurrence of DV] [21].

Outcome variable were defined as a proportion of married women with the experience of one or more acts of physical, sexual, and/or emotional violence by her husband and other in family relationship at any point in their lives were considered to have lifetime experience while if such acts had occurred in last 12 months then it was called past year prevalence or current DV. The dependent variable current DV was taken for inferential analysis i.e. bivariate and multivariate analysis.

#### Independent variables

Data were collected on several levels of variables—individual, family and community which were expected to have association with DV from literature search.

#### Individual level variables

Women’s characteristics: age in years (categorized into age groups); education (formal and no formal education); occupation (housewife/not engaging in any earning work, daily wages labor, service/business). Mental health status was dichotomized as unhealthy (seven or more than seven questions from the SRQ-20 answered affirmatively) and healthy (less than seven questions from the SRQ-20 answered affirmatively) [22].

Husband’s characteristics: age in years (categorized into age groups); employment status (unemployed, unskilled workers, semi-skilled/skilled workers); education (formal and no formal education); alcohol taking habit (often, sometimes/rarely, no).

#### Family level and community level variables

Family Size (less than five members and more than or equals to five members); duration was noted on the basis of number of completed years of marriage. Castes were categorized on the basis of Nepal government system and the respondents were asked whether their husband possess any extramarital relationship. Marriage type was categorized as love marriage and arranged marriage. Respondents were asked if the family annual income from all sources was sufficient for 1 year.

Husband’s controlling behavior was measured as:

1. Keeps her from seeing friends.
2. Restricts her contact with family.
3. Insists on knowing where she is all the times.
4. Gets angry when she talks with other men.

In case of the occurrence of at least one of the above mentioned activities performed by the husband, he was designated as a controlling husband [20].

SPSS 20 version and EPI info 7 were used for statistical analysis. The completed questionnaires were included in this study. The crude association between dependent and independent variables were inspected by Chi square and Fischer exact test at 95 % CI. Variables showing p value of less than 0.20 from bivariate analysis were entered into logistic model for multivariate analysis. Hosmer–Lemeshow test was done to observe the fitness of model and the variables causing poor fit were excluded. Binary logistic regression was applied and adjusted odds ratio (AOR) at 95 % CI was calculated in order to identify the associated factors. Cronbach’s alpha was 0.79 for thirteen items of DV and it was 0.71 for twenty items of SRQ. The obtained value was calculated after pretesting with the sample of 40.

## Results

### Prevalence of different forms of violence

Out of 355 eligible women who completed the study, 38.6 % of the respondents were victims of at least one type of violence ever from various perpetrators with emotional violence being the most common (31 %). It was followed by physical violence (29.6 %) and sexual violence (6.8 %). Nearly one-fourth of the women (23.1 %) reported experiencing violence in the past 12 months with physical violence (15.2 %), sexual violence (2.3 %) and emotional violence (18.3 %) respectively (Table 1).

**Table 1:** Lifetime and current prevalence and frequency of domestic violence (n = 355)

S.N.	Forms of domestic violence^a^	Lifetime	Past 12 months
		Frequency	Frequency
1	Physical violence^a^		
1.1	Pushed her, shook her, or threw something at her	92 (25.9)	50 (14.1)
1.2	Slapped her	87 (24.5)	50 (14.1)
1.3	Twisted her arm or pulled her hair	67 (18.9)	38 (10.7)
1.4	Punched her with his fist or with something that could hurt her	57 (16.1)	27 (7.6)
1.5	Kicked her, dragged her, or beat her up	49 (13.8)	26 (7.3)
1.6	Tried to choke her or burn her on purpose	–	1 (0.3)
1.7	Threatened her or attacked her with a knife, gun, or any other weapon	14 (3.9)	9 (2.5)
*At least one episode of physical violence*		*105* (*29.6*)	*54* (*15.2*)
2	Sexual violence^a^		
2.1	Did your husband physically force you to have sexual intercourse even when you did not want to?	14 (3.9)	8 (2.3)
2.2	Did your husband force you to perform any sexual acts you did not want to?	16 (4.5)	5 (1.4)
2.3	Did you husband did any degrading or humiliating sexual act?	6 (1.7)	6 (5.6)
*At least one episode of sexual violence*		*24* (*6.8*)	*8* (*2.3*)
3	Emotional violence^a^		
3.1	Said or did something to humiliate her in front of others	94 (26.5)	55 (15.5)
3.2	Threatened to hurt or harm her or someone close to her	42 (11.8)	25 (7.0)
3.3	Insulted her or made her feel bad about herself	86 (24.2)	50 (14.1)
*At least one episode of emotional violence*		*110* (*31.0*)	*65* (*18.3*)
*At least one episode of three violence*		*137* (*38.6*)	*82* (*23.1*)

Number in parenthesis indicates percentages

^a^Multiple responses

**Fig. 1:**
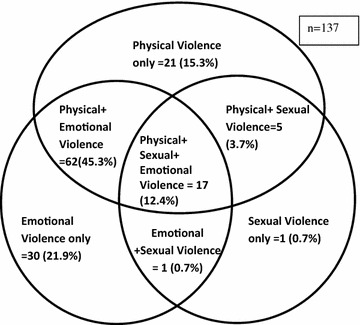
Venn diagram showing different forms of violence

The different forms of violence and their overlapping nature are elucidated in Fig. 1. The most commonly occurring single form was emotional violence (21.9 %) followed by physical violence (15.3 %). It was found that 12.4 % had experienced all three types of violence concurrently (Fig. 1). Victims of DV were further asked open questions like who were their perpetrators, whether they reported the violent episodes or not, place of reporting a nd reasons for not reporting. One in every three, among victims of DV, reported husband as the main perpetrator (70.8 %) followed by mother-in-law (32.8 %) and father-in-law (21.9 %). Perceived factors of violence by women were also explored where half of the respondents (51.1 %) reported alcohol as the prime reason followed by norms supporting violence (43.1 %) which includes male dominated society, women accepting violence, women who perceive wife beating as justified act of husband etc. Among 137 victims of DV, only 19.7 % had reported the violence among which maternal home (33.3 %) was preferred for reporting their sufferings after police (37 %). More than 80 % of the victims didn’t report the episode of violence and when asked for the reason it was found that the most common reason was accepting violence as normal/a part of life (54.5 %).

### Factors associated with DV (Bivariate analysis)

Table 2 represents the association of several individual level variables with current DV. Daily wages labors were at higher risk of violence as compared to women involved in service or small scale business (COR = 4.091, 95 % CI 1.355–12.438). Educational status was dichotomized as no formal education and formal education which was found to be significantly associated (COR = 1.764, 95 % CI 1.006–3.091) with current DV. Mentally unhealthy women were found to be at almost three times more risk for current DV. Having an unemployed husband was found to increase the risk of violence by two times as compared to women having husband involved in semiskilled/skilled works. Women whose husbands drink alcohol often are at more than two folds greater risk of experiencing DV as compared to women whose husband never drink.

**Table 2:** Bivariate association of individual level variables and current domestic violence (n = 355)

Characteristics	Current domestic violence		*p* value	Crude odds ratio (95 % CI)
	Yes	No		
*Women’s characteristics*				
Current age in years				
15–24	17 (23.6)	55 (76.4)	0.893	1.185 (0.513–2.735)
25–44	53 (23.6)	172 (76.4)		1.181 (0.583–2.393)
45–49	12 (20.7)	46 (79.3)		Ref.
Occupation of women				
Housewife/don’t earn	64 (23.7)	206 (76.3)	*0.037*	2.330 (0.950**–**5.713)
Daily wages labor	12 (35.3)	22 (64.7)		*4.091* (*1.355* **–** *12.438*)
Service/small business	6 (11.8)	45 (88.2)		Ref.
Education of women				
No formal education	174 (73.7)	62 (26.3)	*0.046*	*1.764* (*1.006* **–** *3.091*)
Formal education	20 (16.8)	99 (83.2)		Ref.
Mental health status of respondents				
Unhealthy	30 (41.7)	42 (58.3)	<*0.001*	*3.173* (*1.818* **–** *5.537*)
Healthy	52 (18.4)	231 (81.6)		Ref.
*Husbands’ characteristics*				
Current age in years				
≤21	8 (22.6)	4 (77.4)	0.612^a^	0.589 (0.153**–**2.748)
>21	265 (23.6)	78 (76.4)		Ref.
Employment status				
Unemployed	20 (29.9)	47 (70.1)	0.118	*2.083* (*1.015* **–** *4.276*)
Unskilled workers	43 (24.4)	133 (75.6)		1.583 (0.867**–**2.888)
Skilled/semiskilled	19 (17.0)	93 (83.0)		Ref.
Educational status				
No formal education	32 (27.6)	84 (72.4)	0.162	1.440 (0.862**–**2.405)
Formal education	50 (20.9)	189 (79.1)		Ref.
Alcohol consumption				
Often	44 (33.6)	87 (66.4)	*0.001*	*2.621* (*1.463* **–** *4.694*)
Sometimes/rarely	16 (18.2)	72 (81.8)		1.152 (0.567**–**2.338)
No	22 (33.6)	114 (66.4)		Ref.

p value and COR in italics indicates significant

Number in parenthesis indicates percentages

^a^Fisher exact test

From Table 3, it is evident that women who reported of having family income not enough for even 1 year were at more than two folds higher risk of experiencing violence as compared to those women who reported of having income enough for 1 year or more (COR = 2.688, 95 % CI 1.622–4.456). Over one in every five women (20.8 %) reported to have experienced at least one of the controlling behaviors among four from their husbands and they were more than nine times likely to experience current DV than the women having non-controlling husband (COR = 9.379, 95 % CI 5.270–16.691). As compared to women from upper caste, Dalits women were three times more likely to experience violence (COR = 3.11, 95 % CI 1.418–6.826). Women having husband who were married more than once in their lifetime were at almost three folds increased risk of experiencing current DV.

**Table 3:** Bivariate association of family/community level variables and current domestic violence (n = 355)

Characteristics	Current domestic violence		p value	Crude odds ratio (95 % CI)
	Yes	No		
Family size				
>5 members	53 (26.2)	149 (73.8)	0.107	1.521 (0.912**–**2.537)
≤5 members	29 (19.0)	124 (81.0)		Ref.
Duration of marriage (years)				
>10	56 (24.9)	169 (75.1)	0.292	1.325 (0.784**–**2.242)
≤10	26 (20.0)	104 (80.0)		Ref.
Marriage type				
Love marriage	31 (27.2)	83 (72.8)	0.208	1.391 (0.831**–**2.330)
Arrange marriage	51 (21.2)	190 (78.8)		Ref.
Family Income (year)				
Enough for <1	48 (33.8)	94 (66.2)	<*0.001*	*2.688* (*1.622*–*4.456*)
Enough for 1	34 (16.0)	179 (84.0)		Ref.
Husband controlling behavior				
Yes	44 (59.5)	30 (40.5)	<*0.001*	*9.379* (*5.270*–*16.691*)
No	38 (13.5)	243 (86.5)		Ref.
Caste/ethnicity				
Dalits	28 (40.0)	42 (60.0)	*0.002*	*3.11* (*1.418*–*6.826*)
Disadvantaged Janajatis	37 (20.0)	148 (80.0)		1.166 (0.568**–**2.397)
Advantaged Janajatis	5 (15.6)	27 (84.4)		0.864 (0.276**–**2.702)
Upper caste	12 (17.6)	56 (82.4)		Ref.
Involved in polygamous relationship				
Yes	12 (44.4)	15 (55.6)	*0.006*	*2.949* (*1.320–6.587*)
No	70 (21.3)	258 (78.7)		Ref.

p value and COR in italics indicates significant

Number in parenthesis indicates percentages

### Factors associated with DV (multivariate analysis)

Binary logistic regression shows that women having unhealthy mental status were more than two folds at a greater risk of experiencing current DV as compared to women having healthy mental status (AOR = 2.057, 95 % CI 1.056–4.008). Similarly, women who had reported of experiencing at least one of the four husband controlling behaviors were at almost eight times more likely to experience DV compared to women who did not have controlling husbands (AOR = 7.607, 95 % CI 3.980–14.540) (Table 4).

**Table 4:** Mutivariate analysis of current domestic violence (n = 355)

Variables	Categories	p value	AOR	95 % CI
Occupation of women	Housewife/don’t earn	0.583	1.352	0.460**–**3.971
	Daily wage based work	0.333	1.950	0.504**–**7.549
	Service/small business		Ref.	
Educational status of women	No formal education	0.867	1.069	0.489–2.340
	Formal education		Ref.	
Educational status of husband	No formal education	0.190	0.615	0.298–1.271
	Formal education		Ref.	
Employment status of husband	Unemployed	0.679	1.176	0.546–2.533
	Unskilled workers	0.306	0.684	0.331–1.415
	Skilled/semiskilled workers		Ref.	
Alcohol consumption habit of husband	Never		Ref.	
	Sometimes/rarely	0.752	0.867	0.359–2.095
	Often	0.225	1.619	0.744–3.522
Mental health status of respondents	Unhealthy	*0.034*	*2.057*	*1.056*–*4.008*
	Healthy		*Ref.*	
Husband controlling behavior	Yes	<*0.001*	*7.607*	*3.980*–*14.540*
	No		*Ref.*	
Marriage type	Arrange marriage	0.228	0.666	0.344–1.289
	Love marriage		Ref.	
Family size	>5 members	0.192	1.518	0.811–2.840
	≤5 members		Ref.	
Income of the family	Enough for less than 1 year	0.386	1.351	0.684–2.666
	Enough for 1 year		Ref.	
Caste distribution of respondents	Dalits	0.367	1.665	0.550–5.046
	Disadvantaged Janajatis	0.459	0.698	0.270–1.806
	Advantaged Janajatis	0.390	0.546	0.138–2.165
	Upper caste		Ref.	
Polygamous relationship	Yes	0.170	2.026	0.738–5.559
	No		Ref.	

p value and COR in italics indicates significant

## Discussion

Women in Nepal and around the world experiences different forms of violence throughout their lives and not limited to the caste, religion, region to mention few [20, 23]. In this study, lifetime violence obtained was similar to the findings of NDHS 2011 which showed that overall 26 % had ever experienced physical and/or sexual violence [7]. A 2012 study by the Office of the Prime Minister and Council of Ministers indicated that 48 % of respondents had experienced violence and 28 % in the past 12 months [8]. However, in another study in Dang and Surkhet indicated 81 % incidence while 51 % incidence in another study done in Nepal [18, 20]. Wide variation in the prevalence across studies stressed the importance of conducting additional studies on similar topic because of contextual variability of violence. Sexual violence reported in this study is remarkably lower than other similar studies [9] and it might be due to the underreporting in rural settings where people generally don’t complain about male members at any cost because of cultural norms and are scared to expose their personal issues. Comparable findings were found in a study conducted in Kathmandu, where corresponding figures for physical, psychological and sexual violence were 16.7, 35.5 and 3.6 % respectively [14]. Results of our study largely corroborate the findings from Nepal and all over the world such as in India, Pakistan, Ethiopia, Iran etc [24–27].

Few previous studies have investigated the overlaps between different types of violence. The combination of physical and psychological abuse was depicted to be the most commonly occurring violence form, and the similar scenario was seen in other studies as well [28, 29]. These consistencies might be explained as physical violence is often accompanied by psychological attacks, threatening, and controlling behaviors. Our study showed that 12.4 % of women suffered all three forms of violence concurrently which is higher than the findings from rural Vietnam whereas significantly lower proportion found in study by Abeya [28, 29]. The possible explanation for lower occurrence observed in our study is extensively due to the perception of conjugal affairs as being private matter and is not disclosed especially in rural areas. Similar to the findings from other studies, it showed that the violence was multiple in nature and most of the women were subjected to be the victim of more than one type of violence [25, 27, 30].

Control has been acknowledged as a crucial issue in the marital relationships. Strong positive association was observed between DV and perpetration of controlling behaviors by husband [31–33]. This observations point towards the male domination nature of our society. Traditional masculinity is still prevalent in our society and this result highlights the need to engage men and women to challenge norms and bring changes. However there are arguments about controlling behavior, whether it is a contributing factor or part of the violence acts [25]. This possibly indicated male supremacy in marital relation where wives are supposed to obey their husbands and accept everything as a normal male behavior. It compels us to rethink because if women aren’t safe in their homes and from their husband with whom they are supposed to spend their rest of lives, then how can we assure their safety outside home and from others?

Bivariate analysis contributes to evidences that women are subjected to violence because of conventional practices like witchcraft, multi-marriage etc [6, 34]. These findings elucidates the structural, cultural, and social characteristics of our society continue to perpetuate the victimization of women. Mental health status was found to be one of the strongest predictor of DV in accord with previous studies which indicate that it has negative effect on women’s mental health, leading to anxiety, depression, and even suicide [35, 36].

The major perpetrators in this study was husband followed by mother-in-law which is in agreement with other studies [25]. This possibly indicated male supremacy in marital relation where wives are supposed to play submissive role. Furthermore, husbands and mother in law exhibit violent behaviors to control women demonstrating the persistence of dominance power in relationship. Subordinate position of women, limited freedom of expression and their economic dependency lead them to tolerate violence and in male-dominated society like Nepal, the situation is even worse due to deep-rooted norms supporting the violence.

Acceptance of the situation as a part of life was identified by most respondents as a way of responding to violence and they don’t report is to the outsiders considering it as a private and non-interferable matter [8, 18]. GBV is often a hidden problem, as in many countries women fear reprisals for reporting, are unaware of their rights, or lack knowledge of how and where to seek assistance. Though, community mediation centre has been initiated with the objective of dispute resolution at local level, minimal proportion of women had sought assistance from it. When and where women seek care and support if they have suffered from violence depends on many levels like at individual level (women’s autonomy in decision making, unaware about existing policies), at family and community (societal norms around the acceptability and expectation of VAW) and at institutional level (availability of trained manpower, appropriate responses to the need of victims). These factors need to be incorporated while designing interventions.

The major strength of this study is exploration of different forms of violence and inclusion of mental health assessment. Furthermore, it had used standard tools and response rate is also high. Like any study based on self reporting, recall bias may have been associated with disclosure of violent episodes and to minimize this only current prevalence was included in regression analysis. To tackle the issues of underreporting due to the sensitive nature of this issue, maximum efforts were made to ensure privacy and develop trustworthy relationship before data collection and wherever possible, information was cross-checked by asking additional questions. Although this study provides the most comprehensive information to date on the prevalence and associated factors, some potential limitations need to be acknowledged. Social span and biological adaptation according to women’s age alters her personality, mental health and study of those factors was beyond the scope of this study. Being cross sectional in nature, the analysis only provides the evidence of statistical association between the variable but it can’t establish the casual association. Data collection was done only in one VDC due to time and financial constraints so it might be illustrative but not representative.

## Conclusions

This study is inclined towards the growing evidences of prevalence and associated factors of DV. The primary motive and contribution of this study is to show mental health of women as a risk factor of DV. The obtained association between mental health and violence has unbolted future possibilities for various researchers to explore more on this critical issue where longitudinal studies is needed to see temporal relationships. Its prevalence is like an open secret which is as old as the origin of family; however patterns and prevalence vary with time and person. Every associated factor has direct or indirect root into the cultural norms, values of society in this part of world and this revelation can only be a small chunk of it. Despite of several governmental and non-governmental organizations working to eliminate every forms of discrimination against women and enforcement of law against it, various forms are still prevailing in our society and even is in rising trend. Awareness programs regarding where and to whom to seek for help in case of violence need to be conducted and considering the societal context of Nepal, husband and mother-in law should be included in such activities as they can be the most influential person in bringing the change. Formal and informal educational program need to be conducted regarding the act against DV and the concept of community mediation centre. There is a strong need of promising public health strategies include changing attitudes that foster violence and gender inequality, strengthening self-esteem of women and girls and promoting equity in marital relationships.

## Abbreviations

AOR: adjusted odds ratio
BPKIHS: BP Koirala Institute of Health Science
CI: confidence interval
COR: crude odds ratio
FCHV: female community health volunteers
IPV: intimate partner violence
MoFALD: Ministry of Federal Affairs and Local Development
MOHP: Ministry of Health and Population
NDHS: Nepal Demographic Health Survey
SEA: South–East Asia
SRQ-20: self reported questionnaire-twenty items
VAW: violence against women
VDC: Village Development Committee
WHO: World Health Organization
